# Supplementary material for: Solvent induced amyloid polymorphism and the uncovering of the elusive class 3 amyloid topology
Source: Commun Biol. 2024 Aug 9;7:968. doi: 10.1038/s42003-024-06621-8 (PMC11316126; doi:10.1038/s42003-024-06621-8)
Supplement: Supplementary file 2 — Supplementary Information [file 42003_2024_6621_MOESM2_ESM.pdf]

Supplementary Information

Solvent induced amyloid polymorphism and the uncovering of the elusive class 3 amyloid topology

Zsolt Dürvanger[1,2], Fruzsina Bencs[1,3], Dóra K. Menyhárd[1,2], Dániel Horváth[2] and András Perczel[1,2,*]

[1] Laboratory of Structural Chemistry and Biology ELTE Eötvös Loránd University, Pázmány Péter sétány 1/A, H-1117 Budapest, Hungary.

[2] HUN-REN-ELTE Protein Modeling Research Group ELTE Eötvös Loránd University, Pázmány Péter sétány 1/A, H-1117 Budapest, Hungary.

[3] Hevesy György PhD School of Chemistry, ELTE Eötvös Loránd University, Pázmány Péter sétány 1/A, H-1117 Budapest, Hungary.

* perczel.andras@ttk.elte.hu

Supplementary Table 1. The fitted dielectric constant (ε) of the different alcohol-water solvent mixtures at 37°C.

Composition	Dielectric constant (ε) at given temperature [1]						Equation (second degree polynomial, where x= 37°C)	R^2	ε at 37°C
	**20°C**	**30°C**	**40°C**	**50°C**	**60°C**	**80°C**			
**100% H_2O**	80.37	76.73	73.12	69.85	66.62	60.58	$y = 0.0007x^2 – 0.4026x + 88.126$	1	74.19
**10% EtOH**	74.60	-	67.86	64.53	61.49	55.7	$y = 0.0006x^2 – 0.3788x + 81.936$	1	68.74

a

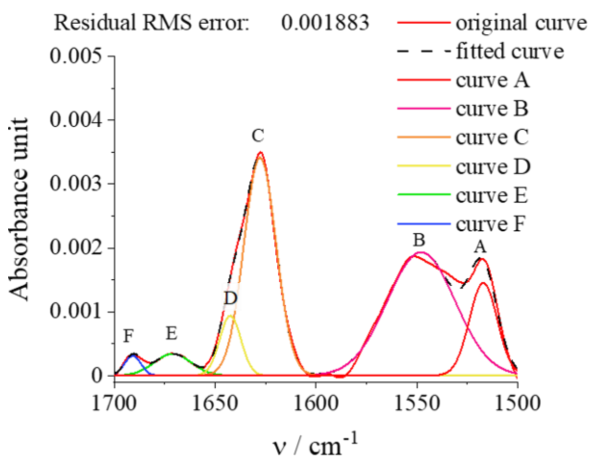

b

Curve	Band position (Gaussian maximum) / cm^{-1}	Assignment
A	1518	Tyr–OH *ν*(CC), *δ*(CH)
B	1548	Amide II
C	1628	Amide I: β-sheet (*main band*)
D	1642	Disordered
E	1671	Gln *ν*(C=O),
F	1690	β-sheet (*side band*)

Supplemenary Figure 1. Assignment of IR bands in the spectrum of LYIQWL recorded in water **a**) The IR spectrum of LYIQWL was recorded after mixing and incubating at 37°C for 6 hours. **b**) Decomposition of the IR spectrum into Gaussian curves and bands assignments. [2,3]

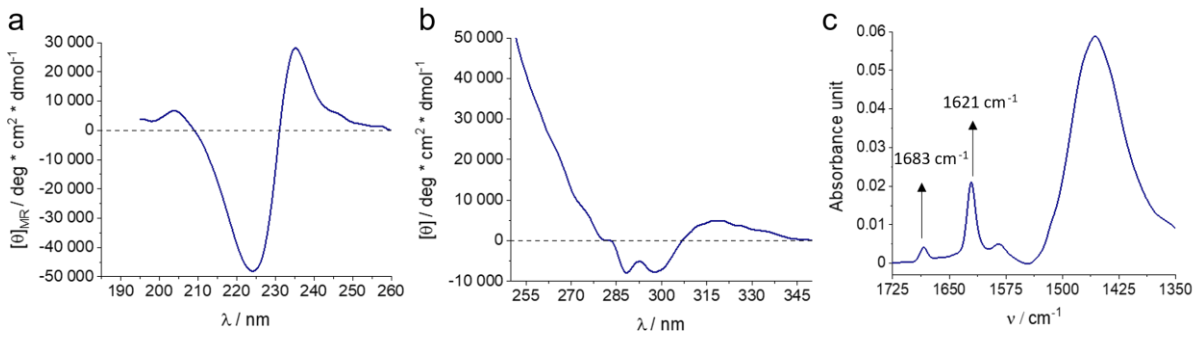

Supplementary Figure 2. **Spectroscopic data of LYIQWL at 4 mg/ml concentration**. Far-UV ECD (**a**), Near-UV ECD (**b**) and FTIR (**c**) spectra of LYIQ**W**L recorded in ^2H_2O. All spectra were acquired from the same stock solution with the following parameters: $c_{(peptide)}$=4 mg/mL, *pH*= 4.8 and *T*=37°C. Panel **a** shows the signal related to the aromatic interaction [4] in the far-UV ECD spectrum, which is the same as that recorded in the case of LYIQ**W**L at 1.5 mg/mL concentration in water at pH=3.8. Based on previously published results, the NMR measurements confirmed that a Trp-Tyr interaction occurs in the E5 miniprotein.[5] The near-UV ECD measurement of the native α-helical secondary structure E5 miniprotein gave a curve similar to panel **b**. In light of these findings, it can be assumed that the

LYIQWL peptide, derived from E5, forms an antiparallel β-sheet structure under these conditions, as the Trp-Tyr interaction can only occur in this case. This is supported by the appearance of a side band characteristic of an antiparallel β-sheet at 1683 cm^{-1} [3] in the FTIR spectra shown in panel **c**.

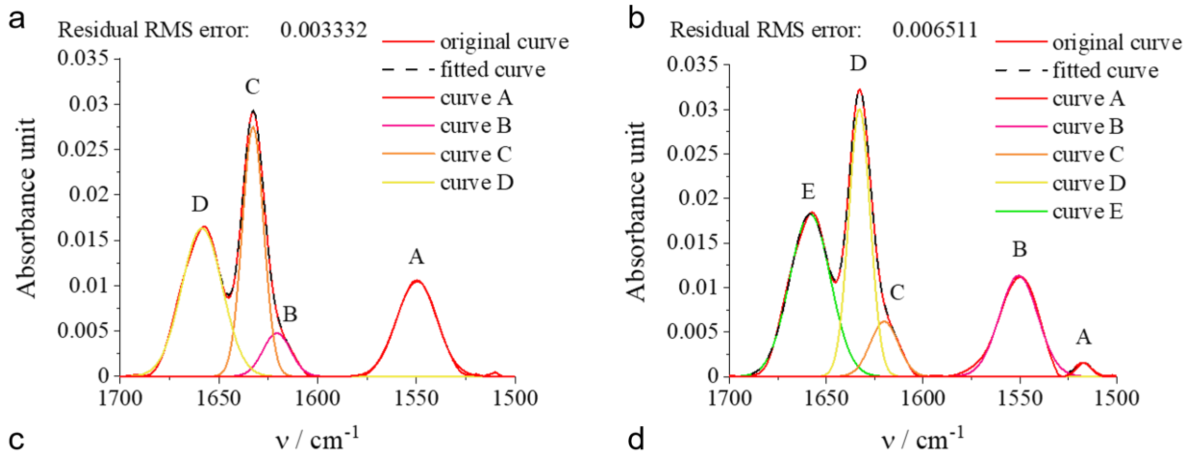

c

Curve	Band position (Gaussian maximum) / cm^{-1}	Vibration
A	1550	Amide II
B	1621	Tyr–OH ν(CC), δ(CH)
C	1633	Amide I : β-sheet
D	1658	Gln and Asn ν(C=O)*

d

Curve	Band position (Gaussian maximum) / cm^{-1}	Vibration
A	1517	Tyr–OH ν(CC), δ(CH)
B	1551	Amide II
C	1620	Tyr–OH ν(CC), δ(CH)
D	1633	Amide I : β-sheet
E	1658	Gln and Asn ν(C=O)*

Supplementary Figure 3. Assignment of the IR bands of LYIQNL. The IR spectra of LYIQNL recorded in water (**a**) and in 10% EtOH containing water (**b**) were deconvoluted into Gaussian curves and bands were assigned (**c** and **d**), respectively. *Presumably, the parallel β-strand arrangement allows both for Ans and Gln residues to form an H-bonded ladder amyloid structure, stabilizing the amide groups of their side chains too. This interaction leads to a decrease from ~1677 cm^{-1} to 1658 cm^{-1} in the frequency associated with the ν(C=O) band of Gln and Asn side chain amide group.[3] This is a further indication that the peptide forms an extended and stable structure in solution. The IR spectra were recorded after a 6-hour incubation time at 37°C and mixing. The assignment of the vibration bands is based on literature data.[2,3]

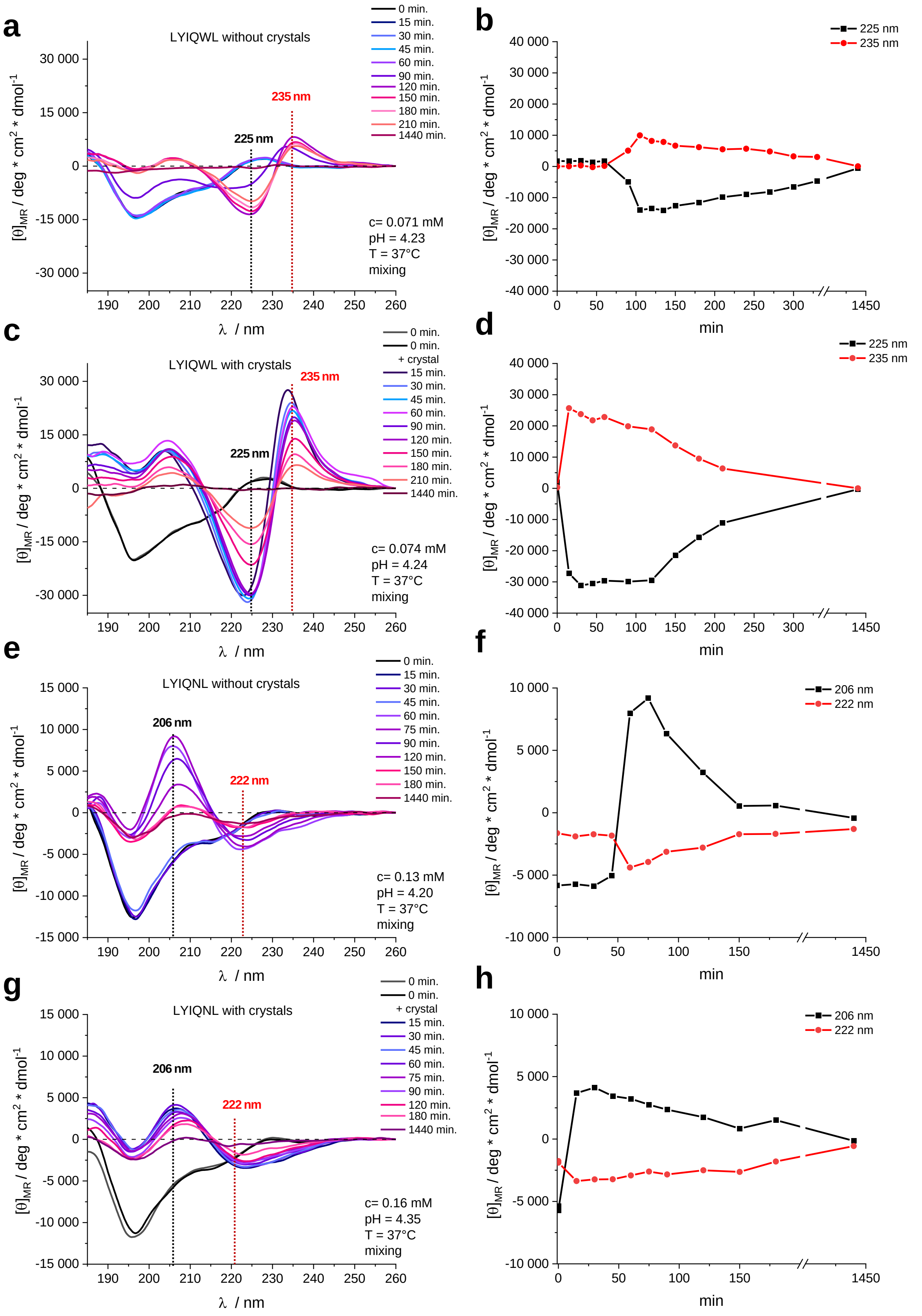

Supplementary Figure 4. Amyloid formation monitored by CD spectroscopy in presence and absence of seed crystals. In the right column, the recorded CD spectra reveal the secondary structure conversions as a function of time for LYIQWL/LYIQNL in the absence (**a** and **e**) and presence (**c** and **g**)

of added seeding crystals, respectively. We highlight the corresponding changes in the CD intensity measured at characteristic wavelengths of LYIQWL (**b,d**) and LYIQNL (**f,h**).

Supplementary Table 2. Polymorphic amyloid crystal structures deposited in the PDB. Sequences with polymorphs belonging to multiple classes are shown in bold.

Sequence	PDB code	Class	Crystallization condition
AALALL	7LUX	7	30 w/v% PEG3000, 0.1M CHES, pH 9.5
	7LTU	7	water, acetonitrile, TFA
GNNQQNY	1YJP	1	water
	2OMM	1	water
**IFAEDV**	5TXJ	7	20 w/v% PEG3350, 0.2M NH_4NO_3
	5TXH	8	20 w/v% PEG4000, 0.1M sodium citrate, pH .6, 20 v/v% isopropanol
KLVFFA	2Y2A	7	0.2M NH_4Ac, 0.1M Bis-Tris, pH 5.5, 45 v/v% MPD
	3OW9	7	30 v/v% Jeffamine M-600, 0.1M MES, pH 6.5, 0.05M CsCl, 1 mM FDDNP
MVGGVV	2OKZ	8	20 v/v% isopropanol, 0.2 M $CaCl_2$, 0.1M NaAc, pH 4.6
	2ONA	8	30 v/v% MPD, 0.02M $CaCl_2$, 0.1M NaAc, pH 4.6
**MVGGVVIA**	2Y3K	2	1.26 M NaH_2PO_4, 0.14M K_2HPO_4, pH 5.6
	2Y3L	7	0.1M Hepes, pH 7.5, 0.5 M Mg formate
**NNQQ**	2OLX	1	0.1M trisodium citrate, pH 5.6, 35 v/v% tert-butanol
	2ONX	4	0.1M trisodium citrate, 20 w/v% PEG4000, 20 v/v% isopropanol, pH 5.6
NVGSNTY	3FTK	1	0.1M Hepes, pH 7.5, 25 v/v% PEG3350
	3FTL	1	0.1M Hepes, pH 7.5, 25 v/v% PEG3350
SSTNVG	3DG1	1	30 v/v% MPD
	3FTR	1	20 w/v% PEG3000, 0.1M Hepes, pH 7.5, 0.2M NaCl
VQIVYK	2ON9	1	0.2M NH_4Ac, 0.1M Hepes, pH 7.5, 45 v/v% MPD
	4NP8	1	14 v/v% isopropanol, 30 v/v% glycerol, 0.14M sodium citrate, 0.07M Hepes, pH 7.5
**LFIEWL**	8ANN	8	30 v/v% acetonitrile, 0.1% TFA
	8ANL	7/8	water
**LYIQWL**	8ANI	8	10 v/v% ethanol, residual TFA
	8ANM	8	water
	8ANG	1	30 v/v% ethanol
	8QWW	4	10 v/v% ethanol, without residual TFA
**LYIQNL**	8QWV	3	0.1M acetate, pH 4.80, ethanol present
	8QWU	4	0.1M acetate, pH 4.80

Supplementary Table 3. Crystallization conditions of LYIQWL and the resulting polymorphic forms. In case of unpublished structures, classes were assigned based on cell parameters being identical to previously solved structures.

Temperature	Concentration (mg/ml)	Conditions	Residual TFA	Class	Result (pdb code)
4 – 37 °C	0.5 – 1.0	water	+	8	(identical to) 8ANM
20 – 37 °C	0.5	5 v/v% ethanol	+	8	identical to 8ANM
20 – 37 °C	0.5 – 0.75	10 v/v% ethanol	+	8	(identical to) 8ANI
4 – 37 °C	0.5 – 1.0	30 v/v% acetonitrile	+	8	8ANH (identical to 8ANI*)
37 °C	0.5	10 v/v% acetone	+	8	identical to 8ANI*
20 – 37 °C	0.5 – 1.0	30 v/v% ethanol	+	1	8ANG
37 °C	0.25 – 0.75	10 v/v% ethanol	-	4	8QWW
4 °C	0.25 – 0.75	10 v/v% ethanol	-	8	identical to 8ANM

* identical arrangement of the peptide chains with different solvent molecules (acetonitrile, ethanol or acetone) at similar positions

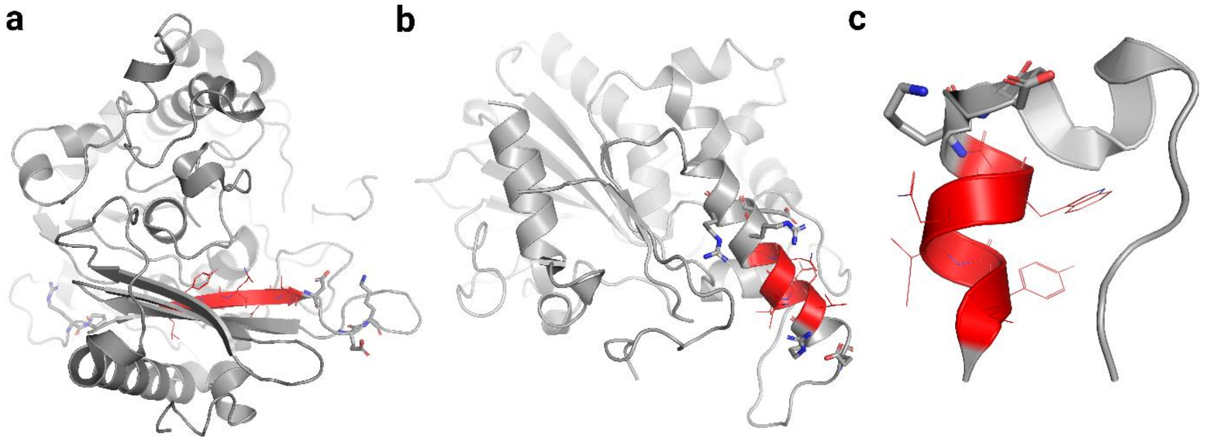

Supplementary Figure 5. Ribbon diagram of proteins of known 3D structure containing the studied primary sequence bits. **a)** *Bacteroides thetaiotaomicron* Endo-4-O-sulfatase (PDB code: 6S21) and **b)** Human Palmitoleoyl-protein carboxylesterase NOTUM (PDB code: 6ZYF) contain the LYIQNL sequence (coloured in red). Gatekeeper residues flanking the amyloidogenic LYIQNL segments are shown as sticks: LYIQNL**D**^{414} in 6S21 and **R**^{361}LYIQNLG**R**^{369}in 6ZYF, respectively. **c)** The Tc5b miniprotein (PDB code: 1L2Y) contains the LYIQWL sequence embedded in an α-helix and followed by two charged residues: LYIQWL**KD**^{9}.

Supplementary Table 4. Analytical characteristics of selected oligopeptides *via* their MS data and retention times.

Primary Sequence	$M_{mono.}$ Calculated[1] (Da)	$M_{mono.}$ Measured[2] (Da)	Retention time[3] (min)
LYIQNL	762.4276	762.43	15.95
LYIQWL	834.4640	834.46	18.85

[1]Calculated monoisotopic molecular mass was determined by https://www.protpi.ch/Calculator/ProteinTool; [2]Exact monoisotopic molecular mass (M_{mono}) was measured on a Bruker AmaZon SL™ Electrospray ionization Ion Trap Mass Spectrometer; [3]Retention time was obtained on Aeris™ 3.6 µm PEPTIDE XB-C18 100Å, 4.6 x 250 mm column, using the gradient: from 5% to 95% B eluent in 30 minutes with a Jasco LC-2000Plus HPLC system. A: H_2O (0.1% TFA), B: MeCN (0.08% TFA); flow rate: 0.9 mL/min.

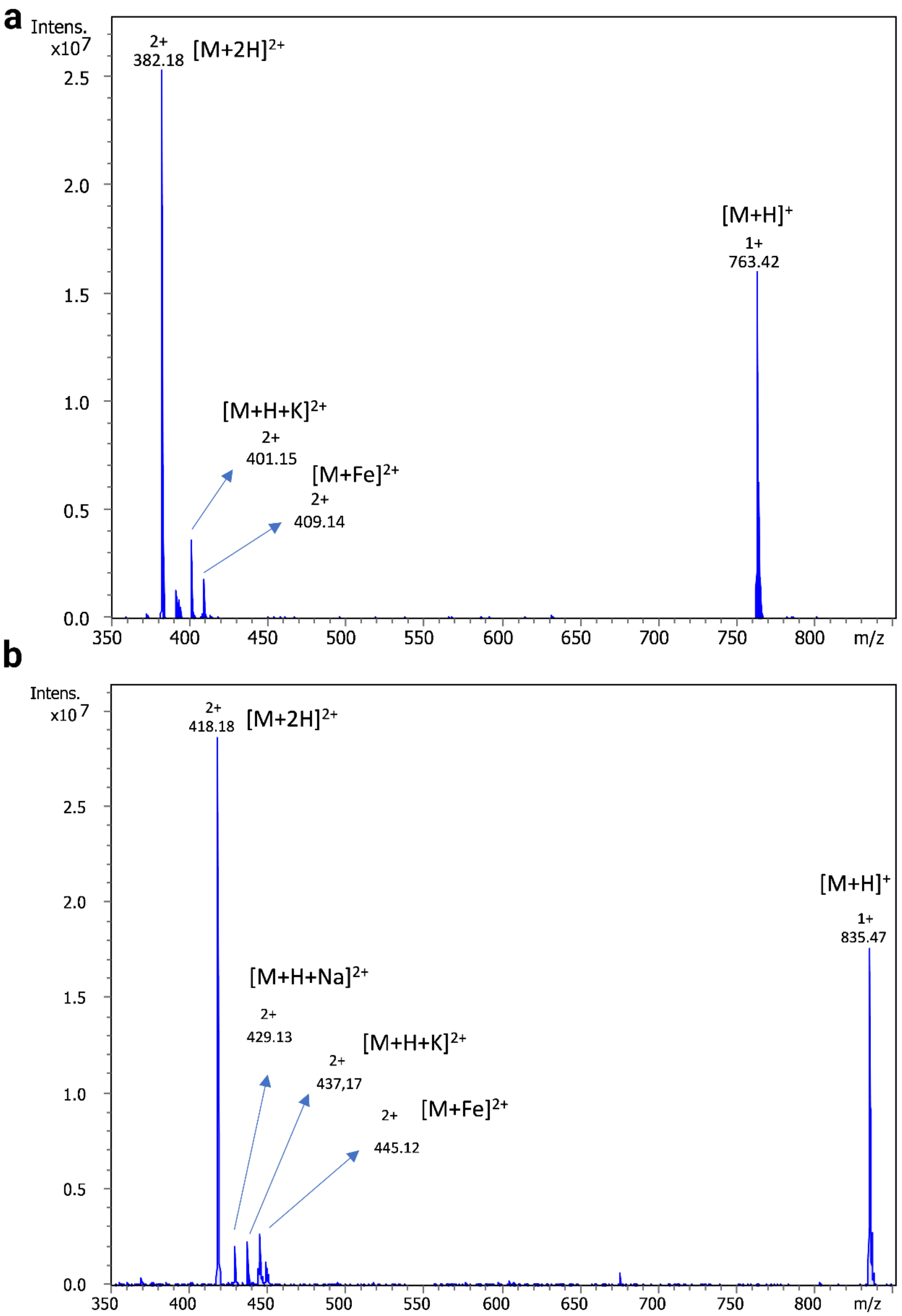

Supplementary Figure 6. MS spectra of the purified LYIQNL (a) and LYIQWL (b) peptides measured on a Bruker AmaZon SL™ Electrospray ionization Ion Trap Mass Spectrometer. **The high voltage applied during electrospray ionization can generate* Fe^{2+} *ions, which are prone to form adducts with hexapeptides.*

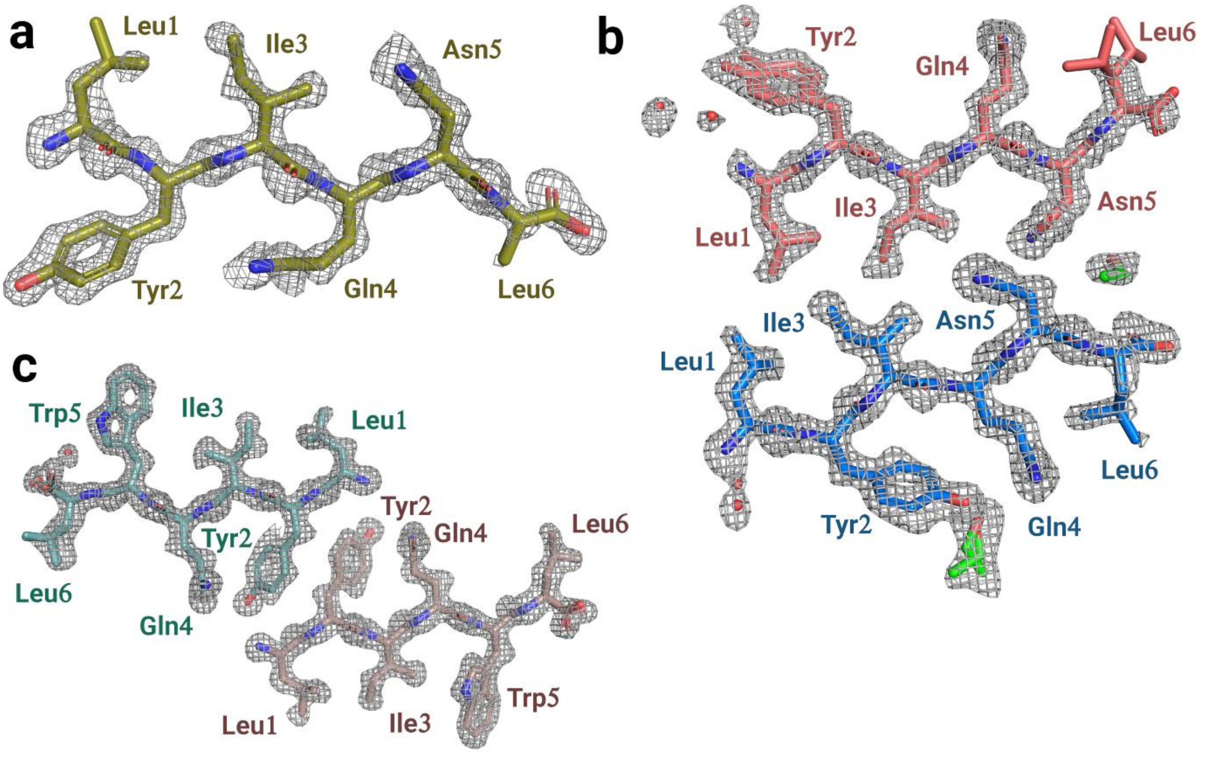

Supplementary Figure 7. Electron density maps around the crystal structures. 2mF_o-DF_c electron density maps around the contents of the asymmetric units of crystals of LYIQNL grown from water (class 4, **a**), from an ethanol – water mixture (class 3, **b**) and LYIQWL grown from 10 v/v% ethanol without the presence of TFA (**c**)

Supplementary references

1. Akerlof, G. Dielectric constants of some organic solvent-water mixtures at various temperatures. *J. Am. Chem. Soc.* **54**, 4125–4139 (1932).
2. Barth, A. The infrared absorption of amino acid side chains. *Progress in Biophysics and Molecular Biology* **74**, 141–173 (2000).
3. Barth, A. Infrared spectroscopy of proteins. *Biochimica et Biophysica Acta (BBA) - Bioenergetics* **1767**, 1073–1101 (2007).
4. Wu, L., McElheny, D., Takekiyo, T. & Keiderling, T. A. Geometry and Efficacy of Cross-Strand Trp/Trp, Trp/Tyr, and Tyr/Tyr Aromatic Interaction in a β-Hairpin Peptide. *Biochemistry* **49**, 4705–4714 (2010).
5. Taricska, N. *et al.* The Route from the Folded to the Amyloid State: Exploring the Potential Energy Surface of a Drug- Like Miniprotein. *Chem. Eur. J.* **26**, 1968–1978 (2020).
