# Supplementary material for: Solvent induced amyloid polymorphism and the uncovering of the elusive class 3 amyloid topology
Source: Commun Biol. 2024 Aug 9;7:968. doi: 10.1038/s42003-024-06621-8 (PMC11316126; doi:10.1038/s42003-024-06621-8)
Supplement: Supplementary file 3 — Description of Additional Supplementary Files [file 42003_2024_6621_MOESM3_ESM.pdf]

Description of Additional Supplementary Files

File name: Supplementary data 1

Description: Source data behind the graphs in the manuscript and the supplementary information file
