# Supplementary material for: Solvent induced amyloid polymorphism and the uncovering of the elusive class 3 amyloid topology
Source: Commun Biol. 2024 Aug 9;7:968. doi: 10.1038/s42003-024-06621-8 (PMC11316126; doi:10.1038/s42003-024-06621-8)
Supplement: Supplementary file 5 — Reporting Summary [file 42003_2024_6621_MOESM5_ESM.pdf]

nature portfolio

Corresponding author(s): Prof. András Perczel

Last updated by author(s): Jul 1, 2024

Reporting Summary

Nature Portfolio wishes to improve the reproducibility of the work that we publish. This form provides structure for consistency and transparency in reporting. For further information on Nature Portfolio policies, see our Editorial Policies and the Editorial Policy Checklist.

Statistics

For all statistical analyses, confirm that the following items are present in the figure legend, table legend, main text, or Methods section.

n/a	Confirmed	
☐	☒	The exact sample size (*n*) for each experimental group/condition, given as a discrete number and unit of measurement
☐	☒	A statement on whether measurements were taken from distinct samples or whether the same sample was measured repeatedly
☒	☐	The statistical test(s) used AND whether they are one- or two-sided<br>*Only common tests should be described solely by name; describe more complex techniques in the Methods section.*
☒	☐	A description of all covariates tested
☒	☐	A description of any assumptions or corrections, such as tests of normality and adjustment for multiple comparisons
☐	☒	A full description of the statistical parameters including central tendency (e.g. means) or other basic estimates (e.g. regression coefficient) AND variation (e.g. standard deviation) or associated estimates of uncertainty (e.g. confidence intervals)
☒	☐	For null hypothesis testing, the test statistic (e.g. *F*, *t*, *r*) with confidence intervals, effect sizes, degrees of freedom and *P* value noted<br>*Give P values as exact values whenever suitable.*
☒	☐	For Bayesian analysis, information on the choice of priors and Markov chain Monte Carlo settings
☒	☐	For hierarchical and complex designs, identification of the appropriate level for tests and full reporting of outcomes
☒	☐	Estimates of effect sizes (e.g. Cohen's *d*, Pearson's *r*), indicating how they were calculated

Our web collection on statistics for biologists contains articles on many of the points above.

Software and code

Policy information about availability of computer code

Data collection	CrysAlisPro v 1.171.42.58a, OPUS 6.0, Jasco Spectra Manager v.2.0, Nanosurf control software C3000 version 3.10.4
Data analysis	Phenix 1.20.1, Coot 0.9.8.5, CCP4 8.0, Jasco Spectra Manager v.2.0, Gwyddion 2.62, Buster 2.10.4, Origin2020b, Excel 2016-2020, accessiblesurfacearea v7.2 (doi: 10.5281/zenodo.6321286), PyMOL 2.40

For manuscripts utilizing custom algorithms or software that are central to the research but not yet described in published literature, software must be made available to editors and reviewers. We strongly encourage code deposition in a community repository (e.g. GitHub). See the Nature Portfolio guidelines for submitting code & software for further information.

Data

Policy information about availability of data

All manuscripts must include a data availability statement. This statement should provide the following information, where applicable:

- Accession codes, unique identifiers, or web links for publicly available datasets
- A description of any restrictions on data availability
- For clinical datasets or third party data, please ensure that the statement adheres to our policy

Novel crystal structures have been deposited in the PDB database under accession codes 8QWV [https://doi.org/10.2210/pdb8QWV/pdb], 8QWU [https://doi.org/10.2210/pdb8QWU/pdb] and 8QWW [https://doi.org/10.2210/pdb8QWW/pdb]. Previously published crystal structures of LYIQWL can be obtained from the PDB database under accession codes 8ANM [https://doi.org/10.2210/pdb8ANM/pdb], 8ANI [https://doi.org/10.2210/pdb8ANI/pdb] and 8ANG [https://

doi.org/10.2210/pdb8ANJ/pdb]. Structures of Bacteroides thetaiotaomicron Endo-4-O-sulfatase and human Palmitoleoyl-protein carboxylesterase NOTUM and Tc5b were obtained from the PDB database with accession codes 6S21 [https://doi.org/10.2210/pdb6S21/pdb], 6ZYF [https://doi.org/10.2210/pdb6ZYF/pdb] and 1L2Y [https://doi.org/10.2210/pdb1L2Y/pdb], respectively. Processed ECD and IR data used for the generation of graphs are provided in the Source Data file. Raw spectroscopic data and unprocessed AFM images will be shared upon request by contacting the corresponding author.

Research involving human participants, their data, or biological material

Policy information about studies with human participants or human data. See also policy information about sex, gender (identity/presentation), and sexual orientation and race, ethnicity and racism.

Reporting on sex and gender	N/A
Reporting on race, ethnicity, or other socially relevant groupings	N/A
Population characteristics	N/A
Recruitment	N/A
Ethics oversight	N/A

Note that full information on the approval of the study protocol must also be provided in the manuscript.

Field-specific reporting

Please select the one below that is the best fit for your research. If you are not sure, read the appropriate sections before making your selection.

☒ Life sciences ☐ Behavioural & social sciences ☐ Ecological, evolutionary & environmental sciences

For a reference copy of the document with all sections, see nature.com/documents/nr-reporting-summary-flat.pdf

Life sciences study design

All studies must disclose on these points even when the disclosure is negative.

Sample size	Statistical methods are not necessary for the determination of sample sizes for structure determination of macromolecules.
Data exclusions	No data were excluded from the analyses.
Replication	Crystallization experiments were carried out under several, highly similar conditions and produced needle-like crystals. Crystals of sufficient quality for diffraction measurements were obtained only from the described conditions. Diffraction measurements were carried out once. ECD and IR measurements were also carried out once.
Randomization	Images were taken from random locations during AFM measurements. Randomization was not necessary for other experimental procedures used in this study.
Blinding	Blinding was not necessary in this study.

Reporting for specific materials, systems and methods

We require information from authors about some types of materials, experimental systems and methods used in many studies. Here, indicate whether each material, system or method listed is relevant to your study. If you are not sure if a list item applies to your research, read the appropriate section before selecting a response.

Materials & experimental systems

n/a	Involved in the study	
☒	☐	Antibodies
☒	☐	Eukaryotic cell lines
☒	☐	Palaeontology and archaeology
☒	☐	Animals and other organisms
☒	☐	Clinical data
☒	☐	Dual use research of concern
☒	☐	Plants

Methods

n/a	Involved in the study	
☒	☐	ChIP-seq
☒	☐	Flow cytometry
☒	☐	MRI-based neuroimaging

Plants

Seed stocks	N/A
Novel plant genotypes	N/A
Authentication	N/A
